# A Mutant of Africa Swine Fever Virus Protein p72 Enhances Antibody Production and Regulates the Production of Cytokines

**DOI:** 10.3390/v17020194

**Published:** 2025-01-30

**Authors:** Mingzhi Li, Yihao Wang, Quansheng Wang, Lingdi Yang, Shiguo Liu, Guangzhi Li, Ziqi Song, Chulu Huang, Lumei Kang, Yanni Zhang, Ting Wang, Lingbao Kong, Sha Li

**Affiliations:** 1 Institute of Pathogenic Microorganism, Jiangxi Agricultural University, Nanchang 330029, China; 18537677196@163.com (M.L.);; 2 Nanchang City Key Laboratory of Animal Virus and Genetic Engineering, Nanchang 330029, China; 3 College of Bioscience and Engineering, Jiangxi Agricultural University, Nanchang 330029, China; 4 College of Animal Science and Technology, Jiangxi Agricultural University, Nanchang 330029, China; 5 Center for Laboratory Animal Science, Nanchang University, Nanchang 330031, China; 6 Jiangxi Province Center for Disease Control and Prevention, Nanchang 330029, China

**Keywords:** ASFV p72, mutant, IFN-γ, HIF1α, AKT

## Abstract

African swine fever virus (ASFV) is a severe threat to the global pig industry, and domestic pigs mostly develop severe clinical manifestations upon viral invasion. Currently, there is no available vaccine against ASFV. Its capsid structural protein p72 is one of the immuno-dominant proteins. In this study, we unexpectedly obtained a p72 mutant protein (p72_∆377–428_) which deleted the aa 377–428 within p72 and had stable and high expression in *E. coli*. Using SWISS-MODEL 1.0 software, the prediction showed that p72_∆377–428_ was quite distinct from the wild-type p72 protein in structure. p72_∆377–428_ induced stronger antibody production in mice on day 42 and 56 post immunization and could recognize ASFV-infected swine sera. p72_∆377–428_ reduced IFN-γ production in the splenocytes from p72_∆377–428_-immunized mice and p72_∆377–428_-treated swine macrophages compared to p72. p72_∆377–428_ also decreased the production of pro-inflammatory cytokine genes, including IL-1β, IL-6, and IL-12, compared to p72 in mice. Further, we found that p72_∆377–428_ reduced the induction of pro-inflammatory cytokine genes by inhibiting AKT phosphorylation and HIF1α expression. Taken together, these findings have implications for immunological function and the corresponding mechanism of ASFV p72, and our study indicates that p72_∆377–428_ could serve as a novel candidate for ASFV vaccines and diagnostic reagents.

## 1. Introduction

ASFV, a dominant threat to domestic pigs, devastates the global swine industry [1]. Although culling infected pigs and increasing biosecurity levels have reduced ASFV transmission heavily, the re-emergence of ASF in Europe demonstrates the urgent need to develop more effective measures to control ASFV [2]. In recent decades, extensive efforts have been made to develop a vaccine against ASFV [3,4,5]. However, the limited knowledge about the function of ASFV proteins and the complex viral composition has delayed this progress [6].

As an icosahedral DNA virus, ASFV contains more than 150 proteins [6,7,8]. Among them, the indispensable capsid structural protein p72 accounts for almost one-third of the total viral mass [2,9]. The p72 protein exhibits strong antigenicity, typically eliciting high antibody titers in ASFV-infected pigs, making it a critical target for the development of serological diagnostics for African swine fever (ASF) [10,11]. Moreover, the p72 protein plays a pivotal role in immunity against ASFV infection and serves as the primary component in the development of nearly all subunit vaccines against ASF [11,12]. As one of the dominant antigens detected in infected pigs, p72 contains multiple antigenic epitopes [13,14,15,16,17]. p72 molecules usually form a trimer with two jelly roll fold domains in each monomer [9,14]. The top of the p72 trimer is a propeller-like structure that extends to the outside of the virus [6] and is most likely the region where the neutralizing epitopes are located [15]. Immediately following viral infection, a strong host response is initiated, and viral proteins stimulate cellular signal transduction and pro-inflammatory cytokine gene expression [18]. Pro-inflammatory cytokines play crucial roles in the promotion of immune and inflammatory responses [19]. Interferon gamma (IFN-γ), a pro-inflammatory cytokine, is primarily known to be produced by T helper cell type 1 (Th1) lymphocytes, CD8 lymphocytes, B cells, NKT cells, and antigen-presenting cells (monocytes, macrophages, and dendritic cells) [20]. It possesses strong antiviral activity, promotes NK cell activity and macrophage classical activation, and orchestrates the activation of the innate immune system [19,20,21,22,23,24,25,26,27,28]. ASFV-specific stimulation of the cells from immune pigs revealed a robust Th1 response characterized by rapid IFN-γ-dependent triggering of an inflammatory response [29]. Considering the correlation between cytokine regulation and ASFV pathogenesis, understanding how ASFV and its proteins regulate these soluble factors will facilitate a more comprehensive understanding of pathogen–host interactions, which in turn will contribute to the current design of safe and effective vaccines or therapeutics against this deadly hemorrhagic viral disease.

Hypoxia and inflammation are inherently linked [30]. Hypoxia-inducible factor-1α (HIF1α) could help cells to obtain enough ATP to survive under hypoxia. In a resting cell, HIF1α is hydroxylated at conserved proline residues by prolyl hydroxylases (PHDs). This hydroxylation allows for HIF1α ubiquitination by the von Hippel–Lindau (VHL) E3 ubiquitin ligase, marking it for rapid proteasomal degradation. PHDs are oxygen-dependent; under normoxic conditions, HIF1α is continuously turned over by means of degradation, resulting in low basal HIF1α levels [30]. Hypoxic conditions result in PHD inhibition and attenuation of HIF1α hydroxylation. In the absence of proteasomal degradation, HIF1α accumulates, translocates to the nucleus, and increases transcription of hypoxia response element (HRE)-containing genes [31]. HIF1α binds the core consensus sequence 5′-(A/G)CGTG-3′ within the HRE present in many genes [32,33,34]. HRE-containing genes mainly encode proteins that allow for cell adaptation to hypoxic environments [30]. There is a whole range of immune-related genes that have HREs in their promoters such as IFN-γ and IL-1β, which are important cytokines in inflammatory responses [35,36]. Therefore, there is a close relationship between HIF1α and pro-inflammatory cytokines.

In this study, we unexpectedly obtained a mutant ASFV p72 protein, p72_∆377–428_, which contained the deletion of aa 377–428 and had stable and high expression in *E. coli*. This deletion was predicted to convert the native p72 trimer from a compacted tertiary configuration to a loose configuration based on the Swiss-Model analysis. p72_∆377–428_ elicited stronger humoral immunity in mice but reduced the secretion of IFN-γ and other pro-inflammatory cytokines including IL-1β, IL-6, and IL-12 in mice or porcine macrophage lines compared to p72. We further observed that p72_∆377–428_ reduced the induction of pro-inflammatory cytokine-related genes by decreasing HIF1α via the inhibition of AKT phosphorylation.

## 2. Materials and Methods

### 2.1. Principal Reagents

High-fidelity DNA polymerase (Cat: R010A), restriction enzymes (Cat: 1605), and T4 DNA ligase (Cat: 2011A) were purchased from Takara. Antibodies against His_6_ tags (Cat: 66005-1-Ig, Clone: No. 1B7G5), HRP-goat anti-murine IgG (Cat: SA00001-1), and HRP-goat anti-swine IgG (Cat: 6050-05) were from Proteintech. Antibodies against HA (Cat: TA100012) and FLAG (Cat: TA50011, Clone: OTI4C5) tags were from ORIGENE. Antibodies against AKT (Cat: WL0003b) were obtained from Wanleibio. Rabbit polyclonal antibodies against Phospho-AKT1/2/3 (Ser473) (Cat: AF0016) were obtained from Affinity. BL21 (DE3) chemically competent cells (Cat: CD601-02) and TransZol Up (Cat: ET111-01) reagent were obtained from Trans. The bacterial lysis buffer (Cat:12650-88-3) was from the Sangon biotechnology company. Protein Markers (Cat: 20350ES72), Hieff Trans^®^ Liposomal Transfection Reagent (Cat: 40802ES03), and Hifair^®^ V one-step RT-gDNA digestion SuperMix for qPCR (Cat: 11141ES10) were from YEASEN. ChamQ Universal SYBR qPCR Master Mix (Cat: Q711-02) was from Vazyme. Amicon^®^ Ultra-15 Centrifugal Filters (Cat: UFC9030) and PVDF Membranes (Cat: ISEQ00010) were from Millipore. Fetal bovine serum (Cat: 10100147) was from Gibco. The Ni-NTA column (Cat: 30210) was from Qiagen. Freund’s complete (Cat: F5881) and incomplete (Cat: F5506) adjuvants (CFA and IFA) were from Sigma. IPTG (Cat: I8070), DMEM medium (Cat:12100), and RPMI 1640 medium (Cat: 31800) were from Solarbio. TMB Chromogen Solution (Cat: P0209) and Protein A + G Agarose (Cat: P2012) was from Beyotime. BCA Protein Assay Kit (Cat: CW0014) was from cwbio. Mouse IFN-γ (Cat: 551866) and IL-4 (Cat: 555232) cytokine detection kits were from BD Biosciences. Mouse IL-1β (Cat: SYP-M0026), IL-6 (Cat: SYP-M0327), and IL-12 (Cat: SYP-M0189) cytokine detection kits were from Upingbio. Porcine IFN-γ ELISA KIT (Cat: CD-JK71030) was from chundubio. MonPro ECL Ultrasensitive Substrate Pro (Cat: PW30701S) was from Monad.

### 2.2. Cell Culture, Bacterial Strains, Mice, and Porcine Sera

HEK293T cells were purchased from the China center for type culture collection. An HEK 293T cell line was cultured in DMEM medium supplemented with 10% heated-inactivated fetal bovine serum at 37 °C in the presence of 5% CO_2_. The porcine macrophage cell line 3D4/21 (Cat: CRL-2843) and Salmonella Typhimurium 14,028 were from ATCC. In addition, 3D4/21 cells were maintained in RPMI 1640 medium supplemented with 10% heated-inactivated fetal bovine serum at 37 °C in the presence of 5% CO_2_, while Salmonella was cultured in LB broth at 37 °C. Four-week-old *Kunming* female mice were obtained from Jiangxi Traditional Chinese Medicine University (License No. SCXK 2018-0003). Porcine sera were obtained from Jiangxi swine farms in China.

### 2.3. Prediction of Antigenic Epitopes and Three-Dimensional Structure of p72 and p72_∆377–428_ Proteins

The prediction of B-cell epitopes and T-cell epitopes and the creation of three-dimensional structures of p72 and p72_∆377–428_ proteins were performed using the predictor of Bepipred 2.0 or the IEDB recommended on the IEDB website and the SWISS-MODEL server, respectively. The relevant information of the software is shown in Supplementary Table S1.

### 2.4. Constructs of Plasmid

The p72 protein-encoding gene B646L was synthesized according to the sequence of the ASFV strain of China/2018/AnhuiXCGQ (GenBank No. MK128995) and was separately cloned into the pET-28a vector with a His_6_-tag at each end. After confirming the orientation and correctness of the gene, we transformed the resultant plasmid (pET-p72) (GenBank No. OL804292) into an *E. coli* BL21 (DE3) strain and examined its expression under the induction of IPTG. Unexpectedly, we found that a clone of *E. coli* BL21 (DE3) stably expressed a protein with a reduced molecular weight compared to full-length p72. We investigated the finding by isolating the plasmid from the *E. coli* BL21 (DE3) clone. Gene sequencing revealed that the protein with a reduced molecular weight compared to p72 was expressed from a mutant p72 gene lacking aa 377–428 (p72_∆377–428_) (GenBank No. OL804293), which might result from gene recombination in *E. coli*. Using a similar strategy, we constructed the plasmids pCAGGS-HA-p72, pCAGGS-HA-p72_∆377–428_, pCAGGS-HA-HIF1α, and pCDNA3.1-Flag-HIF1α to express the recombinant proteins in the eukaryotic system. The HIF1α gene was obtained from 3D4/21 cells by RT-PCR. The primers are shown in Supplementary Table S2. All constructs were confirmed by DNA sequencing. pET-p72 and pET-p72_∆377–428_ sequence data have been submitted to NCBI GenBank under accession numbers No. OL804292 and No. OL804293.

### 2.5. Expression of Recombinant ASFV p72 and p72_∆377–428_ Proteins

A single clone of *E. coli* containing either pET-p72 or pET-p72_∆377–428_ was cultured in LB selective medium overnight. The next day, bacterial culture was added to fresh LB medium supplemented with 50 ug/mL kanamycin at a ratio of 1:100. When the cell density (OD_600nm_) reached 0.6, different concentrations of IPTG were added to induce protein expression. After induction, cells were harvested by centrifugation, washed with ice-cold PBS, and resuspended in lysis buffer (Cat: 1126750–88-3, Sangon Biotechnology company, Shanghai, China). The bacterial paste was stored at −80 °C for further processing. Bacterial lysis was carried out via sonication of thawed bacterial paste with 200 cycles of two working seconds and four resting seconds on ice. The sonicated samples were centrifuged. The supernatant and the pellet (inclusion bodies) were harvested separately for further examination by 10% SDS-PAGE gel.

### 2.6. Optimization of Recombinant Protein Expression at a Single-Parameter Level

The productivity of recombinant p72 and p72_∆377–428_ was evaluated at a variety of IPTG concentrations (0.1, 0.5, 1.0, 1.5, or 2.0 mM), temperatures (20, 25, 32, 37, or 40 °C), and durations (2, 3, 4, 5, or 6 h). These parameters were individually investigated. The yields of the recombinant proteins were evaluated using 10% SDS-PAGE gel.

### 2.7. Purification of Recombinant Proteins

Purifications of the recombinant proteins were conducted as described previously [37]. Briefly, after being sonicated, bacterial inclusion bodies were obtained by centrifugation and re-suspended in an 8 M urea buffer. The resulting supernatant was applied to a Ni-NTA column. Purified proteins were eluted with buffers containing 500 mM of imidazole. The elution was renaturated using dialysis, concentrated by using Ultra-15 Centrifugal Filters, and then stored at −80 °C for further use.

### 2.8. Immunoblots

Immunoblots were performed as described previously [38]. Briefly, cells were washed with PBS and lysed with RIPA Lysis Buffer. The lysates and protein samples were mixed with SDS sample loading buffer, boiled for 10 min, separated by SDS-PAGE, and transferred to polyvinylidene fluoride membranes (Millipore). After blocking, the membranes were probed with the primary antibodies, followed by incubation with horseradish peroxidase-conjugated secondary antibody. The target proteins were visualized by using MonPro ECL Ultrasensitive Substrate Pro (Cat: PW30501, Monad, Suzhou, China).

### 2.9. Mouse Immunization

Four-week-old Kunming female mice were randomly divided into the control, p72, or p72_∆377–428_ group. Mice were intraperitoneally (i.p.) immunized with either PBS (control) or each of the recombinant proteins (1mg/kg body weight) in the presence of complete Freund’s adjuvant (CFA) for the first injection. In the following boosts, incomplete Freund’s adjuvant (IFA) replaced CFA for protein immunization. The whole process lasted eight weeks, with a two-week interval between immunizations. Blood was harvested from the tail vein weekly. Murine sera were used to examine the secretions of antibodies and cytokines. At the endpoint of the assay, mice were sacrificed, and spleens were harvested for the preparation of single-cell suspensions.

### 2.10. ELISA

ELISA plates were coated with 2 μg/mL of pure p72 or p72_∆377–428_ protein dissolved in coating buffer at 4 °C overnight. After being blocked with 10% skimmed milk followed by extensive washes, sera from mice immunized with PBS (negative control) or the recombinant protein (p72 or p72_∆377–428_) were added to the plate. HRP-conjugated goat anti-mouse IgG antibody served as the second antibody. Subsequently, the substrate was added, and the absorbance of each well was measured with a Bio-Rad microplate reader at a wavelength of 450nm. The negative control sera readings were considered for the calculation of the cutoff values (cutoff value = mean OD values of the negative control + 2 × SD). The endpoint titer is defined as the reciprocal of the highest dilution of a serum that gives a reading above the cutoff value [37].

To examine the recognition of the recombinant proteins by ASFV-infected swine sera (inactivated at 60 °C for 30 min) [39], we adopted swine sera as the primary antibody and goat anti-swine IgG antibody as the secondary antibody. For IFN-γ, IL-1β, IL-4, IL-6, and IL-12 detection, murine splenocytes were seeded in 96-well plates at 1 × 10^6^ cells/well. After stimulating these cells with various concentrations of the cognate recombinant proteins for 48 h at 37 °C in 5% CO_2_, we harvested the supernatant and examined the secretions of IFN-γ, IL-1β, IL-4, IL-6, and IL-12 according to the manufacturer’s recommendations. Splenocytes from PBS-immunized mice functioned as a negative control. The levels of IFN-γ in the sera of the immunized mice were also examined.

### 2.11. Transfection

Briefly, 293T or 3D4/21 cells were seeded in 6-well plates at 2.5 × 10^5^ cells/well or 24-well plates at 1 × 10^5^ cells/well the day before transfection. pCAGGS-HA-p72, pCAGGS-HA-p72_∆377–428_, pCAGGS-HA-HIF1α, or pCDNA3.1-Flag-HIF1α were transfected into the corresponding cells. Then, 48 h post-transfection, the expressions of the recombinant proteins were examined by immunoblot. Cells transfected with an empty vector (pCAGGS-HA) functioned as a negative control.

### 2.12. Salmonella Typhimurium Infection

Salmonella Typhimurium infection was performed as described previously [40,41]. Salmonella Typhimurium grew in LB broth overnight at 37 °C with shaking at 180 rpm. The next day, the overnight bacterial culture was transferred into fresh LB medium at 1:100 and continued to grow until reaching an OD_600_ of 0.6. Bacteria were pelleted, washed with PBS, and re-suspended in RPMI medium at 2 × 10^6^ CFUs/mL. Moreover, 3D4/21 cells expressing the recombinant proteins were seeded in 24-well plates at 2 × 10^5^ cells/well followed by infection with 1ml of Salmonella Typhimurium suspension at a multiplicity of infection of 10. After incubation for 30 min at 37 °C in 5% CO_2_, the infected cells were washed three times with PBS and supplemented with fresh RPMI medium containing 100 μg/mL gentamicin for 3 h to remove extracellular bacteria. The monolayers were supplemented with RPMI medium containing 20 μg/mL gentamicin for the rest of the experiment. After 24 h, the supernatant was harvested for IFN-γ detection. Cells were lysed with 1% Triton X-100 solution. The bacterial loads of Salmonella Typhimurium were enumerated on LB agar plates with 100 μg/mL streptomycin.

### 2.13. qRT-PCR

To evaluate the transcriptional levels of (IFN-γ, IL-1β, IL-4, IL-6, IL-10, IL-12, HIF1α, PDK1, RPS6, and AKT) in the splenocytes from immunized mice or 293T cells treated with recombinant proteins, we prepared a single splenocyte suspension as described previously. Total RNA was extracted from these splenocytes with TRIzol reagent following the supplier’s instructions. cDNA synthesis was performed according to the manufacturer’s protocols. RT-qPCR was developed in a QuantStudio 5 Real-Time PCR system (Applied Biosystems). Each RT-qPCR was conducted in triplicate in 20 μL as the total reaction volume, containing 10 μL of Universal SYBR Green Master mix, 1 μL of cDNA, and a pair of gene-specific primers. The expression levels of transcripts were normalized to those of the profilin1 or β-actin gene. The fold change in the expression of each target gene was estimated using the 2^−ΔΔCt^ method. All of the real-time qPCR primers are shown in Supplementary Table S3.

### 2.14. RNA-Seq

RNA-seq was performed to compare gene expression profiles between pCAGGS-HA-p72-transfected 293T cells and pCAGGS-HA (empty vector)-transfected 293T cells over a 24 h time period. One randomly chosen sample for each group was used to prepare an RNA-seq library. Total RNA from each individual sample was extracted using Trizol reagent (Invitrogen, Waltham, MA, USA). The total RNA was qualified and quantified using a Nano Drop and Agilent 2100 bioanalyzer (Thermo Fisher Scientific, Waltham, MA, USA). Subsequently, mRNA was isolated using poly-T oligo-attached magnetic beads and then randomly fragmented in fragment buffer at the appropriate temperature. The first-strand cDNA was synthesized using random hexamers, followed by second-strand cDNA synthesis. Afterwards, A-Tailing Mix and RNA Index Adapters were added by incubating the end repair. The cDNA fragments were PCR-amplified and purified by using Ampure XP beads. The products were validated on the Agilent Technologies 2100 bioanalyzer (Thermo Fisher Scientific, Waltham, MA, USA) for quality control. The final cDNA libraries were sequenced on the DNBSEQ-T7 platform (MGI, Shenzhen, China).

### 2.15. Differential Expression and KEGG Enrichment Analysis of Genes

Differential expression analysis was performed using the DEGSeq R package (1.12.0) for a random sampling model. The resulting *p*-values were adjusted using Benjamini and Hochberg’s approach for controlling the false discovery rate. Genes with an adjusted *p*-value < 0.05 and fold change > 2 according to DEGSeq were designated as differentially expressed. KOBAS 3.0 software was used to test the statistical enrichment of differentially expressed genes in KEGG pathways.

### 2.16. Immunoprecipitation

Cells were washed with ice-cold PBS and suspended in RIPA buffer supplemented with 1mM PMSF. Cell lysates were sonicated for 10 min and then incubated for 30 min at 4 °C, followed by centrifugation at 14,000× *g* for 10 min. The supernatants were immunoprecipitated with anti-Flag or anti-HA antibody in the presence of protein A-agarose beads (Beyotime). The immunocomplexes with the beads were collected through centrifugation at 800× *g* for 30 s and then washed four times with lysis buffer. The proteins binding to the beads were boiled with SDS sample loading buffer and then subjected to SDS-PAGE.

### 2.17. Accession Number

The RNA-Seq raw data have been submitted to the NCBI Sequence Read Archive (SRA) under accession number PRJNA1058003 and include HEK 293T cells.

### 2.18. Statistical Analysis

The data were analyzed using GraphPad Prism 9.0 software to determine statistical significance. Intergroup statistical differences were calculated with Student’s *t*-test. *p* < 0.05 was considered statistically significant.

## 3. Results

### 3.1. Expression and Purification of p72 and p72_∆377–428_ Proteins

**Figure 1:**
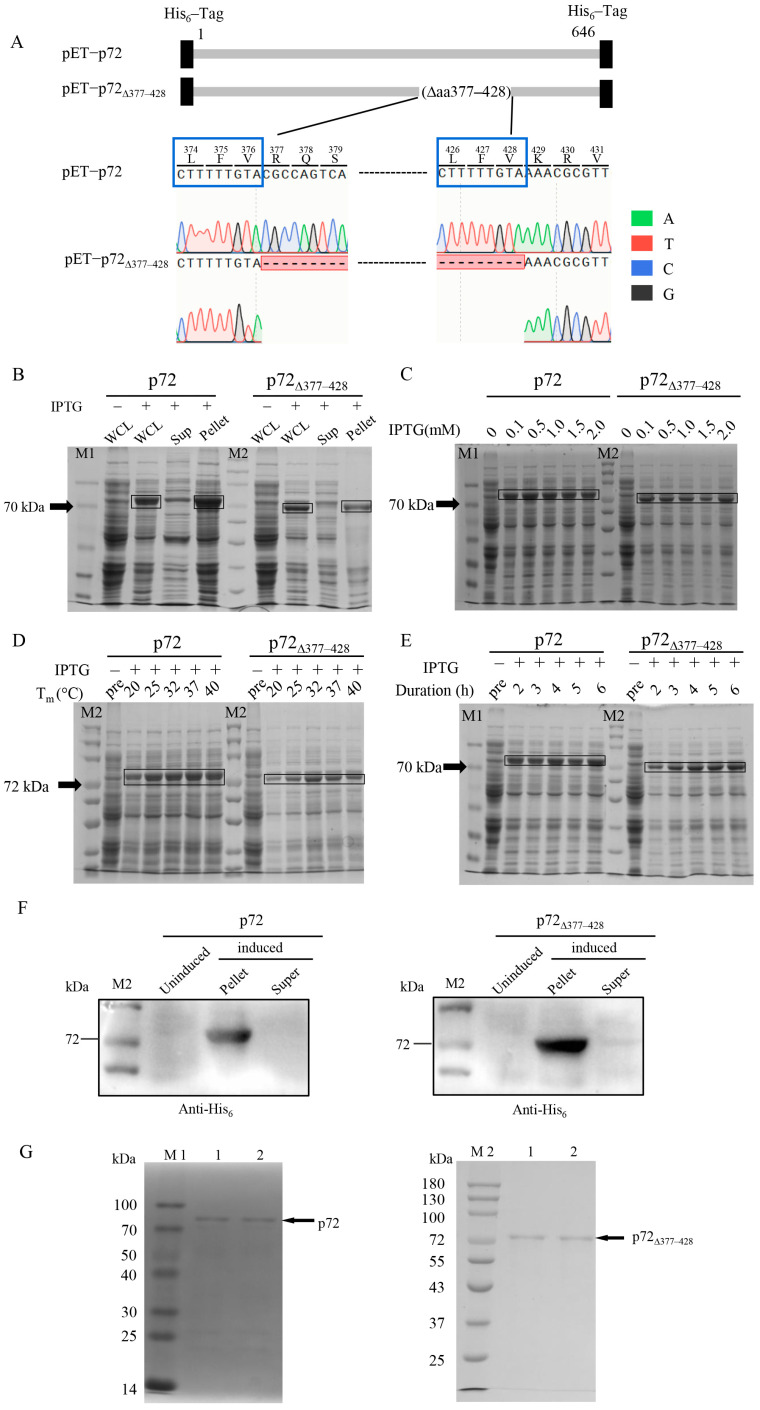
The expression of recombinant p72 and p72_∆377–428_ proteins. (**A**) A diagram and sequencing of recombinant constructs pET-p72 and pET-p72_∆377–428_. (**B**–**E**) The recombinant p72 and p72_∆377–428_ proteins were analyzed on 10% SDS-PAGE gels and visualized with coomassie blue staining. (**B**) Expression analysis of p72 and p72_∆377–428_ by SDS-PAGE. The induced expression conditions for all samples were 1.0 mM IPTG, 4 h, and 37 °C. The data are representative of three independent assays. The contribution of IPTG concentration, temperature, and induction duration to the expressions of p72 and p72_∆377–428_. (**C**–**E**) SDS-PAGE analysis of the effects of IPTG concentration (**C**), induction temperature (**D**), and induced time (**E**) on the protein yields. (**C**) Conditions of 37 °C, 4 h, and various concentrations of IPTG; (**D**) conditions of 1.0 mM IPTG, 4 h, and different temperatures; (**E**) conditions of 37 °C, 1.0 mM IPTG, and different induction times. WCL: whole-cell lysate; Sup: supernatant; pre: pre-induction. (**F**) Identification of p72 and p72_∆377–428_ by immunoblot with an anti-6*His antibody; (**G**) SDS-PAGE analysis of purified p72 (left panel) and p72_∆377–428_ (right panel) proteins. M1/M2: 100kD/180kD protein molecular weight standard. Lane 1/2: purified proteins.

Gene-coding p72 was cloned into the plasmid pET-28a and the resultant plasmid (Figure 1A) was transformed into the *E. coli* BL21 (DE3) strain for expression. Unexpectedly, we found that a clone of *E. coli* BL21 (DE3) stably expressed a protein with a reduced molecular weight compared to full-length p72 (Figure 1B). We investigated the finding by isolating the plasmid from the *E. coli* BL21 (DE3) clone. Gene sequencing revealed that the protein with a reduced molecular weight compared to p72 was expressed from a mutant p72 gene lacking aa 377–428 (p72_∆377–428_) (Figure 1A). p72_∆377–428_ is a mutant form of the ASFV p72 protein, characterized by the deletion of 52 amino acids in the region from 377 to 428, corresponding to a loss of 156 base pairs within the nucleotide sequence from position 1129 to 1284. The sequence “CTTTTTGTA” is a repeated motif found at nucleotide positions 1120–1128 and 1276–1284 within the p72 gene (B646L) (Figure 1A, blue frame). We isolated two distinct clones from the same LB agar plate. Given that the same plasmid (pET-p72) was used for transformation, the likelihood of PCR error amplification resulting in deletions is relatively low. The recombinant system was retained in BL21 (DE3) [42]. We hypothesized that the observed variability may be attributed to recombinase activity within *E coli* BL21 (DE3). Significant levels of the recombinant proteins p72 and p72_∆377–428_ were detected after IPTG induction (Figure 1B). To achieve high yields of the recombinant proteins, we screened the growth parameters at a single level to identify the appropriate operating condition for each protein (Figure 1C–E). According to the results of single-factor analysis, the expression conditions used in subsequent experiments for p72 and p72_∆377–428_ proteins were 0.5 mM IPTG, 37 °C, 2 h, and 0.1 mM IPTG, 32 °C, 4 h, respectively.

Because each recombinant gene had His_6_-tags at both ends, we employed immunoblotting with an anti-His_6_ antibody to identify the recombinant proteins. As shown in Figure 1F, decent bands were observed in IPTG-induced pellets but none were detected in the induced supernatant or un-induced samples. The recombinant proteins were purified using the Ni-NTA column. The entire elution through the Ni-NTA column was collected and subjected to 10% SDS-PAGE. Intensive bands above 70 kDa were detected in the elution containing 500 mM of imidazole for both proteins with purities above 90% (Figure 1G). Interestingly, we found that p72_∆377–428_ exhibited more stable expression in *E. coli* BL21 (DE3) than p72 in our experiments. Therefore, we focus on p72_∆377–428_ in the following studies.

### 3.2. Epitope Analysis and Structural Prediction of the Mutant p72 Protein

**Figure 2:**
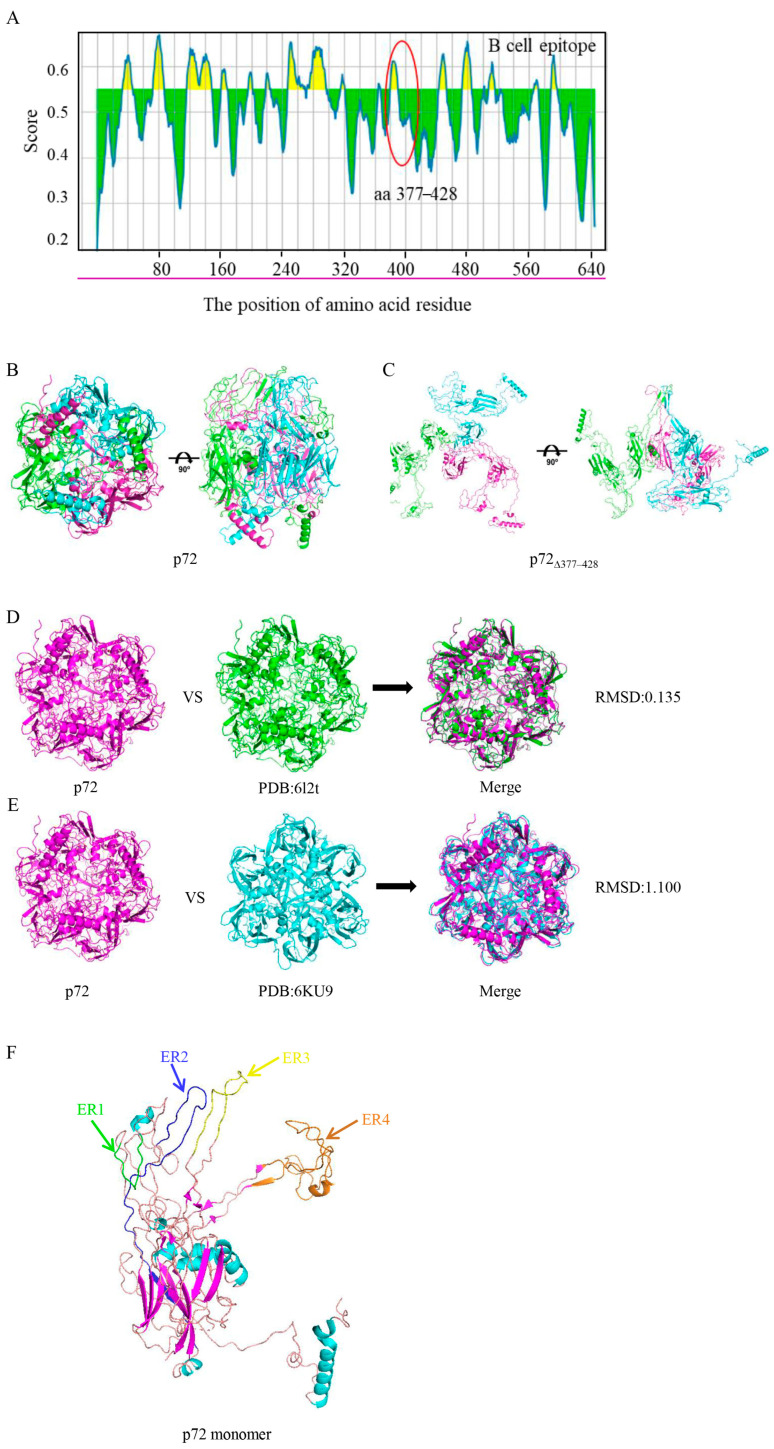
The prediction of antigenic epitopes and the spatial structure of p72 and p72_∆377–428_ proteins. (**A**) The prediction of B-cell potential antigenic epitopes using BepiPred 2.0 with a threshold value ≥ 0.55 (purple line). The region highlighted with the red ellipse is the location of residues 377–428. (**B**,**C**) Ribbon diagrams of the structure of the trimer spikes for p72 (**B**) and p72_∆377–428_. Red, yellow, and blue represent three identical monomers, respectively. (**C**) proteins. The protein monomer is separately represented with azure, pink, and green. (**D**,**E**) The two experimentally determined crystal structures, 6l2t (**D**) and 6KU9 (**E**), were compared with the predicted model. (**F**) Structural analysis of the monomer for p72. The four ER (exposed regions) are marked with green (ER1), indigo (ER2), yellow (ER3), and brown (ER4).

To forecast the linear B-cell epitopes, the p72 protein sequence was subjected to the software of Bepipred 2.0 with a cutoff value of 0.55 (corresponding to a specificity threshold of 82%). The predicted structures of B-cell epitopes related to p72 and p72_∆377–428_ are shown in Supplementary Tables S4 and S5. In total, 20 potential B-cell epitopes were predicted, some of which had already been identified and described in the literature [13,15,17]. One predicted B-cell epitope (RFIAGRPSRRNIRF) at aa 380–393 was missing in p72_∆377–428_ (Figure 2A). Meanwhile, we adopted NetMHCpan EL4.1 on the IEDB to screen T-cell epitopes of the p72 protein and aa 377–428. The IEDB currently recommends making selections based on a percentile rank ≤ 1% for each (MHC allele, length) combination to cover most of the immune responses [43,44]. With H-2D^d^ as the representative MHC molecule, seventy-four and nine epitopes were predicted for the p72 protein and its region of aa 377–428, respectively (Supplementary Table S6). Meanwhile, fifty-seven and four epitopes were predicted for the p72 protein and its region of aa 377–428, with SLA-0101 as the representative MHC molecule (Supplementary Table S7). The common T-cell epitope of aa 377–428 in two predictions is “VTPEIHNL”, which is located in aa 419–426.

SWISS-MODEL (https://swissmodel.expasy.org, accessed on 1 September 2024.) was employed to predict the three-dimensional structures of the p72 and p72_∆377–428_ proteins. The resultant models showed that three p72 molecules formed a stable trimeric spike with a screw propeller-like structure on the top (Figure 2B). Though the p72_∆377–428_ protein was predicted to preserve the trimeric topology, the loss of aa 377–428 resulted in a change from a “compact” state to an “open” state (Figure 2C). Root Mean Square Deviation (RMSD) can be used to evaluate the structural similarity of proteins; the smaller the RMSD value, the closer the crystal and predicted structures are to each other [45,46]. The RMSD of two proteins with a similar structure is usually less than 2.0. Two experimentally determined crystal structures (PDB:6l2t and 6KU9) of p72 [9,14] were similar to the predicted structure, with RMSD values of 0.135 and 1.100, respectively (Figure 2D–E). Therefore, the prediction models for p72 and p72_∆377–428_ are reliable. The crown of the p72 capsomer is formed by ER1 (aa 125–136) and its neighboring ER4 (aa 241–307) and orients toward the outside of the capsid. β-strands from ER2 (aa 378–393) and ER3 (aa 502–523) within the same subunit constitute a four-stranded β-sheet that shapes the head of the p72 capsomer, and ER2 surrounded by ER1, ER3, and ER4 may link these three ERs (Figure 2F). Therefore, p72_∆377–428_ without ER2 could form an “open” structure.

### 3.3. p72_∆377–428_ Elicits Stronger Humoral Immune Response than p72

**Figure 3:**
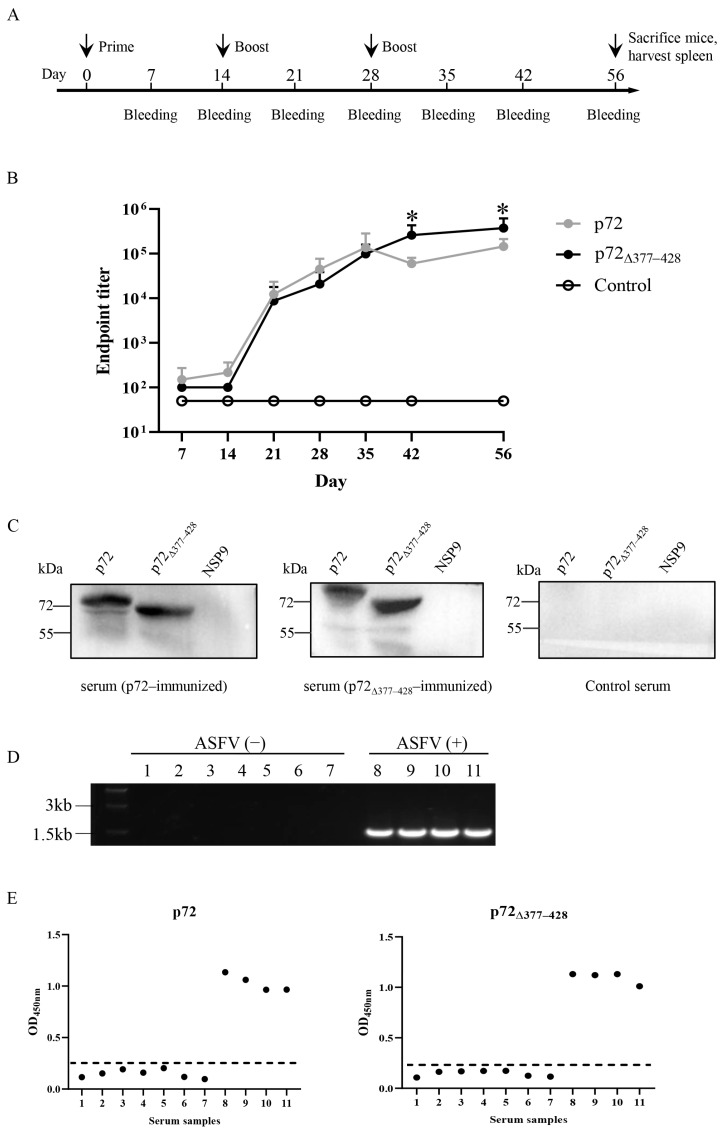
The time course of IgG titers against the recombinant p72 and p72_∆377–428_ proteins and the antigenicity of the recombinant proteins. (**A**) A schematic diagram of the animal experiment design and schedule. Four-week-old Km female mice (*n* = 6–13) were intraperitoneally immunized with each recombinant protein three times at 14-day intervals. At the indicated time points, antisera harvested from immunized mice were subjected to ELISA. (**B**) Humoral responses were induced by the recombinant p72 and p72_∆377–428_ proteins. Antisera were subjected to ELISA to detect p72 and p72_∆377–428_ antibodies. (**C**) The antigenic specificity of the recombinant p72 and p72_∆377–428_ proteins was examined with immunoblotting. Representative images from three independent experiments are shown. Control serum: serum of mice receiving PBS. NSP9: PEDV MBP-NSP9, a non-structural protein from porcine epidemic diarrhea virus. (**D**) PCR was used to screen whether the pig serum came from pigs infected with ASFV. The presence of p72 chaperon-encoding gene (B602L) was used as the readout of ASFV infection. 1–7: irrelevant virus-infected sera; 8–11: ASFV-infected sera. (**E**) Comparison of p72-coated ELISA with p72_∆377–428_-coated ELISA. The recombinant p72 (left panel) and p72_∆377–428_ (right panel) proteins could be recognized by African swine fever-positive sera (*n* = 11). Each dot represents a sample, and the dashed line indicates the cutoff value. African swine fever-positive sera or negative sera served as the primary antibody. HRP-conjugated goat anti-swine IgG functioned as the secondary antibody. Student’s *t*-test was adopted to evaluate the intergroup difference. Error bars indicate standard deviations (SDs) of the means. *: *p* < 0.05.

Antibodies are critical in the protection against viruses. We immunized the mice with the recombinant proteins and examined specific antibody secretions (Figure 3A). Initiation immunization of the recombinant proteins failed to induce robust IgG targeting of the immunogens (Figure 3B). Yet the first boost of either recombinant protein triggered elegant antibody secretion with the titers of 1:12267 for p72 and 1:8667 for p72_∆377–428_ at 21 days (Figure 3B). The extra boost did further upregulate the antibody titers; the p72_∆377–428_ protein induced specific IgG secretion with stronger kinetics, with titers of 1:59733 for p72 and 1:260267 for p72_∆377–428_ at 42 days and titers of 1:145067 for p72 and 1:375467 for p72_∆377–428_ at 56 days (Figure 3B). To examine the immunogenicity of recombinant proteins, immunoblotting was also performed with the sera from protein-immunized mice. *E. coli*-expressed PEDV NSP9 was used as a negative control [47]. Recombinant protein-immunized murine sera specifically detected the corresponding proteins, but not the control protein. As a negative control, the serum from mice treated with PBS did not recognize any recombinant proteins (Figure 3C). To further verify the immunogenicity of recombinant proteins, ASFV-positive swine sera were used for the ELISA assay. Eleven samples of swine sera were obtained from pig farms. PCR amplification using p72-specific primers indicated that seven samples were ASFV-negative and four samples were ASFV-positive (Figure 3D). As expected, the recombinant p72 and p72_∆377–428_ proteins only responded to four ASFV-positive samples (Figure 3E).

### 3.4. p72_∆377–428_ Exhibits Weak Induction Effects on IFN-γ Secretion

**Figure 4:**
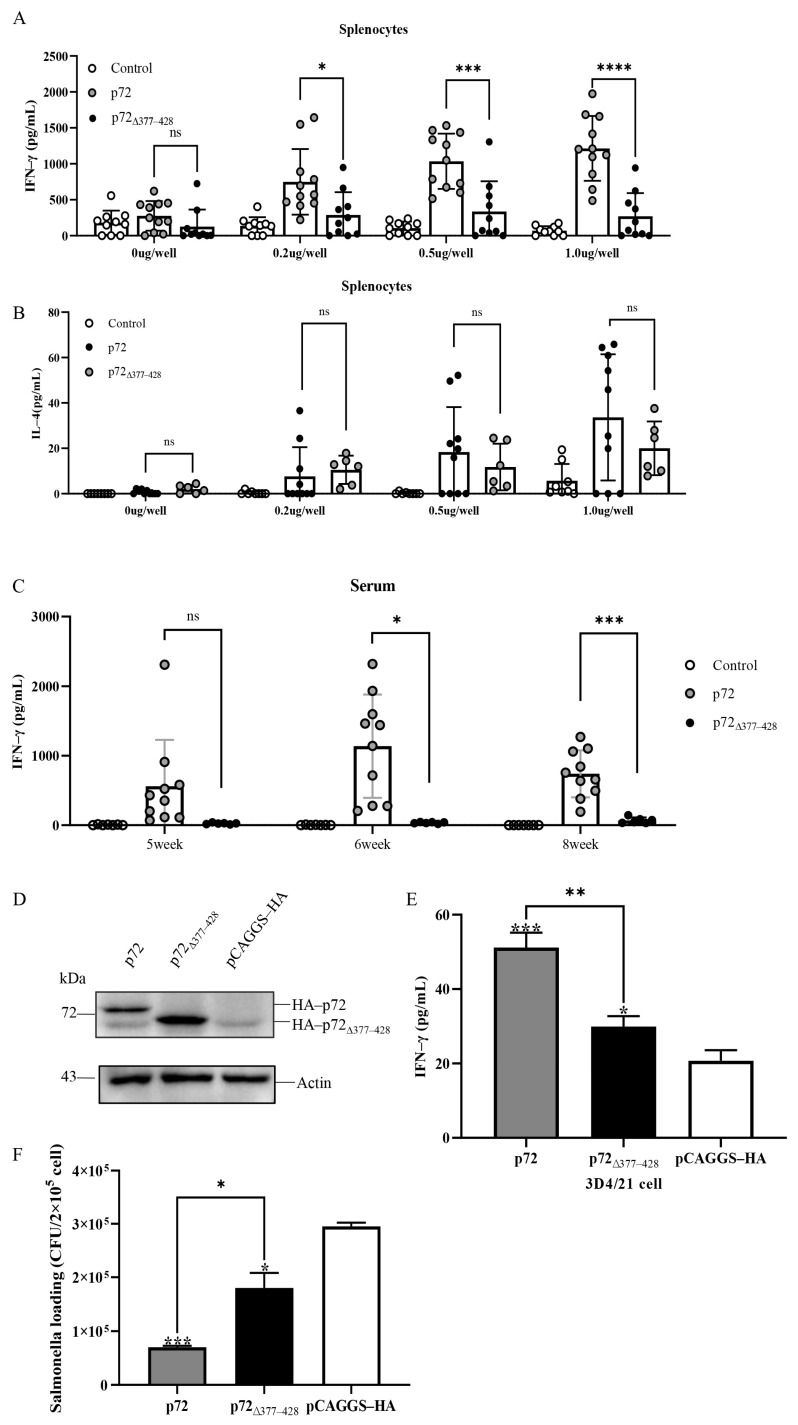
The difference in cellular immunity induced by recombinant proteins p72 and p72_∆377–428_. (**A**,**B**) Splenocytes were harvested from immunized mice and incubated with 0 μg, 0.2 μg, 0.5 μg, and 1 μg of each protein at 10^6^ cells/well. ELISA assays were performed to examine the secretion of IFN-γ (**A**) and IL-4 (**B**) cytokines in the supernatant. (**C**) The detection of IFN-γ in sera of mice immunized with 5-fold diluted after 5, 6, and 8 weeks by ELISA. (**D**) The 3D4/21 cells were transfected with plasmid expressing p72 or p72_∆377–428_. Empty vector-transfected 3D4/21 cells were used as negative controls. After 24 h, the cells were subjected to immunoblot analysis using an HA or actin antibody. Representative images from three independent experiments are shown. Identification of p72 or p72_∆377–428_ expression in 3D4/21 cells by immunoblot. (**E**) The 3D4/21 cells expressing p72 or p72_∆377–428_ were infected with *salmonella* (MOI = 10). Twenty-four hours later, the supernatant of the cell culture was collected to determine the content of IFN-γ. (**F**) The 3D4/21 cells expressing p72 or p72_∆377–428_ bacterial burdens infected with *salmonella*. Student’s *t*-test was adopted to evaluate the intergroup difference. Error bars indicate standard deviations (SDs) of the means. ns: not significant; *: *p* < 0.05; **: *p* < 0.01; ***: *p* < 0.001; ****: *p* < 0.0001.

Cytokine secretions served as readouts of cellular immune responses. Splenocytes derived from the immunized mice were cultured in vitro for 48 h in the context of the appropriate immunogens. IFN-γ and IL-4 secretions in the co-culture were examined by ELISA. IFN-γ secretions from unstimulated isolated splenocytes from p72- and p72_∆377–428_-immunized mice were comparable. Upon stimulation with the cognate immunogens, splenocytes from p72-immunized mice exhibited elevated IFN-γ secretions, which were noticeably higher than those from p72_∆377–428_-immunized mice (Figure 4A). In contrast, IL-4 secretions had no significant difference between p72 immunization and p72_∆377–428_ immunization groups (Figure 4B). Similarly, the sera from p72-immunized mice had more IFN-γ than those from p72_∆377–428_-immunized mice (Figure 4C). Considering that monocyte–macrophages are the main target cells of ASFV, porcine macrophage lines (3D4/21) and primary porcine alveolar macrophages can be induced to secrete IFN- γ [48,49,50]. To further investigate the role of p72 and p72_∆377–428_ in cytokine secretion, we transfected swine 3D4/21 cells with the pCAGGS-HA-p72 or pCAGGS-HA-p72_∆377–428_ plasmid. As expected, the transfected cell lines expressed elegant levels of p72 or p72_∆377–428_ (Figure 4D). Then, we examined IFN-γ production in these cells upon infection with *Salmonella Typhimurium*. The bacterial infection induced p72-expressing cells to secret more IFN-γ productions than p72_∆377–428_-expressing ones (Figure 4E). Consistently, the bacterial burden in p72-expressing 3D4/21 cells was significantly lower than that in p72_∆377–428_-expressing cells (Figure 4F). These results indicate that the p72 protein contributes to the IFN-γ secretion in vivo and in vitro, and the deletion of aa 377–428 within it reduces IFN-γ induction.

### 3.5. p72_∆377–428_ Exhibits Weak Induction Effects on Multiple Pro-Inflammatory Cytokines

**Figure 5:**
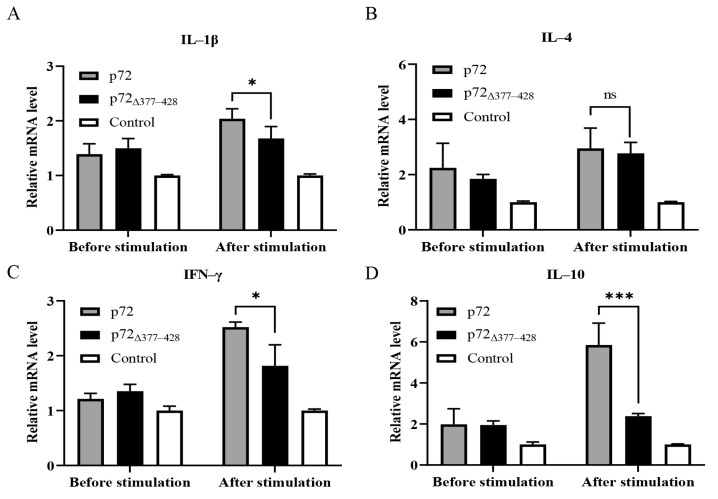

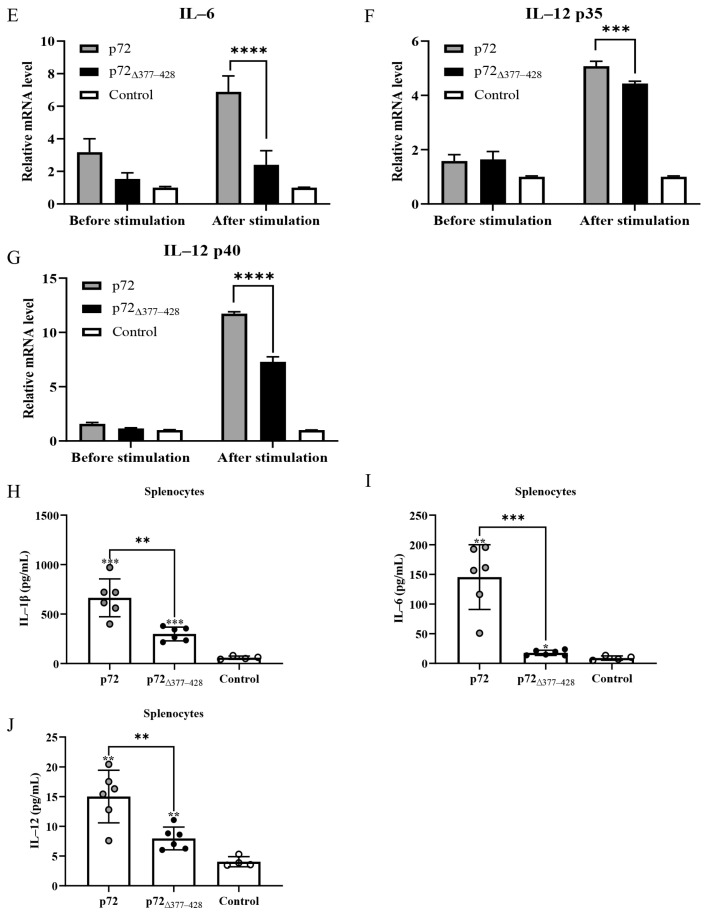
Cytokines induced by immunization of the recombinant proteins p72 and p72_∆377–428_. Splenocytes from mice at 4 weeks after the last immunization were isolated. (**A**–**G**) The RNA of splenocytes was extracted and subjected to real-time PCR to detect the expression level of cytokines IL-1β (**A**), IL-4 (**B**), IFN-γ (**C**), IL-10 (**D**), IL-6 (**E**), IL-12 p35 (**F**), and IL-12 p40 (**G**). (**H**–**J**) The detection of IL-1β (**H**), IL-6 (**I**), and IL-12 (**J**) induced by p72 and p72_∆377–428_ proteins. Splenocytes were harvested from immunized mice and incubated with 1 μg of each protein at 10^6^ cells/well. ELISA assays were performed to examine the secretion of cytokines in the supernatant. The control group was immunized with PBS. Error bars indicate standard deviations (SDs) of the means. ns: not significant; *: *p* < 0.05; **: *p* < 0.01; ***: *p* < 0.001; ****: *p* < 0.0001.

Since p72_∆377–428_ reduced the secretion of IFN-γ compared to p72, we investigated whether p72_∆377–428_ affected the expression of pro-inflammatory cytokines. Therefore, we performed qRT-PCR assays to examine the transcription levels of Th1 (IL-1β, IFN-γ, IL-6, and IL-12) and Th2 (IL-4 and IL-10) cytokines in mouse splenocytes upon immunization with different proteins. Compared with the p72-immunized group, the p72_∆377–428_ -immunized group elicited a lower level of IL-1β, IFN-γ, IL-6, IL-10, and IL-12 (Figure 5A–G). To further evaluate the expression of pro-inflammatory cytokines, after splenocytes were harvested from mice immunized with the p72 or p72_∆377–428_ protein and stimulated by purified proteins in vitro, the secretion of IL-1β, IL-6, and IL-12 from the cell supernatant was examined by ELISA. As shown in Figure 5H–J, p72_∆377–428_ triggered reduced secretions of IL-1β, IL-6, and IL-12 compared with p72. Taken together, these findings revealed that the deletion of aa 377–428 within p72 reduces the induction of pro-inflammatory cytokines in mice.

### 3.6. aa 377–428 of p72 Participates in the Regulation of Pro-Inflammatory Cytokines via the HIF1α Pathway

**Figure 6:**
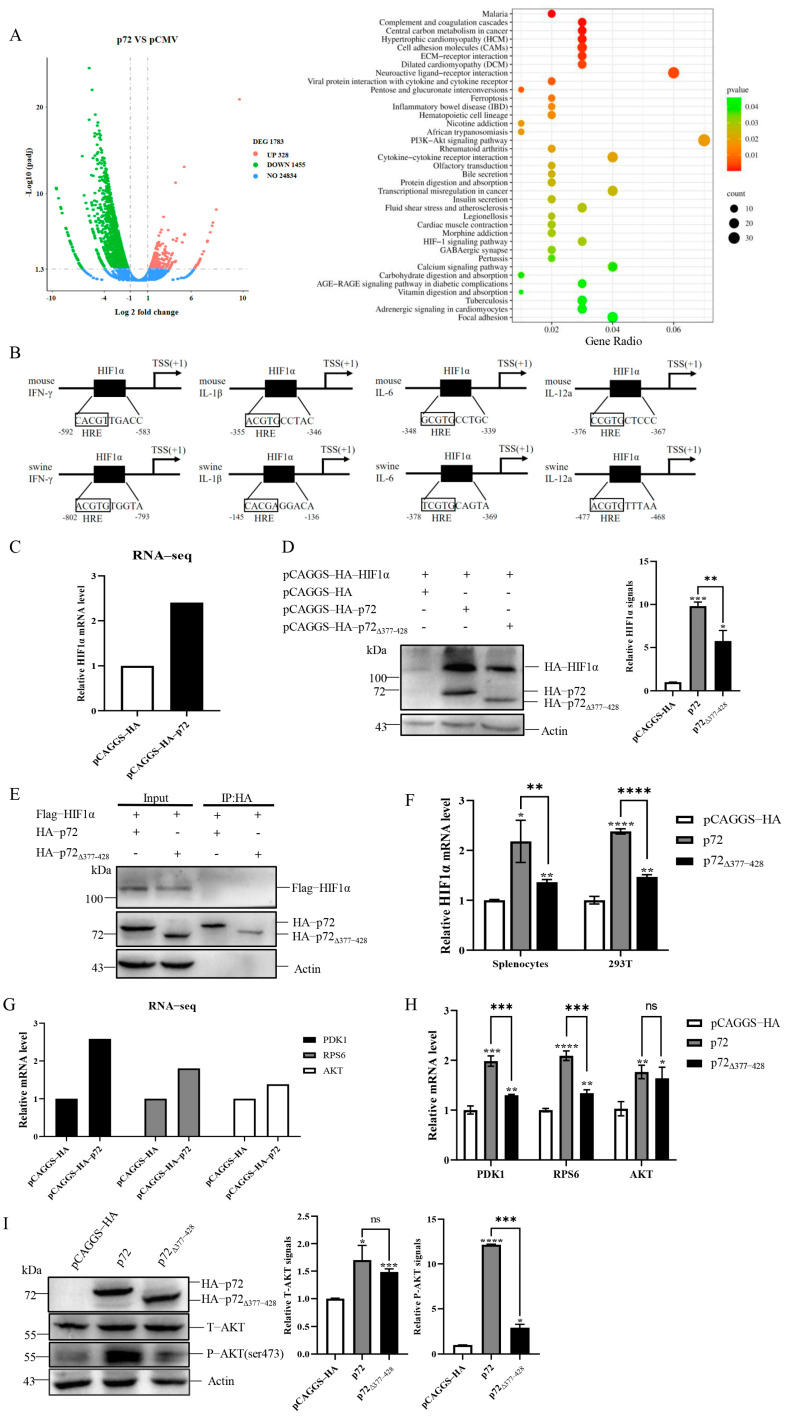
p72_∆377–428_ reduced the induction of cytokine storm-related genes by decreasing HIF1α via the inhibition of AKT phosphorylation. (**A**) The pathways regulated by p72 expression in 293T cells are identified by mRNA profiling. Volcano plots of differently expressed genes in p72-expressing 293T cells (left panel) compared to pCAGGS-HA transfection controls based on RNA-seq. The red dots denote upregulated genes. The green dots denote downregulated genes. The blue dots denote genes with no significant change. Enrichment analysis of pathways in p72-expressing 293T cells by KOBAS software (right panel). (**B**) The schematic structure of the IFN-γ, IL-1β, IL-6, and IL-12 promoter regions of mouse and swine. The transcription start site (TSS) is denoted by a black arrow. The HIF1α binding site (HRE) is shown by a black box in the sequence. HRE: hypoxia response element. (**C**) Analysis of the mRNA level of HIF1α in pCAGGS- or p72-expressing 293T cells by RNA-seq. (**D**,**E**) The 293T cells were transfected with the HIF1α-containing plasmid, as well as the recombinant plasmid expressing either p72 or p72_∆377–428_. Twenty-four hours post-transfection, cell lysates were immunoprecipitated with the appropriate antibody. (**D**) Immunoblot of HIF1α in 293T with overexpression of p72 or p72_∆377–428_. Left panel: Representative images. Right panel: Quantitation data (**E**) The resulting immunoprecipitates (IP) and whole-cell lysates used for immunoprecipitation (Input) were examined by an immunoblot assay using an anti-FLAG antibody or an anti-HA antibody. The interactions between HIF1α and p72/p72_∆377–428_ were detected. (**F**) Analysis of the mRNA level of HIF1α in mice splenocytes and 293T cells by qRT-PCR. (**G**) Analysis of the mRNA level of PDK1, RPS6, and AKT in pCAGGS- or p72-expressing 293T cells by RNA-seq. (**H**) Analysis of the mRNA level of PDK1, RPS6, and AKT in 293T cells with overexpression of p72 or p72_∆377–428_ by qRT-PCR. (**I**) Immunoblot assay of total AKT and phosphorylated AKT in 293T cells with overexpression of p72 or p72_∆377–428_. Left panel: Representative images. Right panel: Quantitation data. Representative images from three independent experiments are shown. Error bars indicate standard deviations (SDs) of the means. ns: not significant; *: *p* < 0.05; **: *p* < 0.01; ***: *p* < 0.001; ****: *p* < 0.0001.

Since we have demonstrated that p72_∆377–428_ reduced the production of pro-inflammatory cytokines including IFN-γ compared to p72, the question of how p72_∆377–428_ caused these effects arose. To address this question, the cellular transcriptome change caused by p72 was analyzed by HTSeq. A total of 1783 genes exhibited statistically significant changes (fold change > 2 and q value < 0.05) (Figure 6A, left panel). The statistical enrichment of differentially expressed genes showed that HIF1α and PI3K/AKT were regulated by p72 (Figure 6A, right panel). HIF1α has been reported to bind the core consensus sequence 5′-(A/G)CGTG-3′ within the hypoxia response element (HRE) present in many genes including pro-inflammatory cytokines [32,33,34]. We observed the potential HREs in the core promoter of pro-inflammatory cytokines including IFN-γ, IL-1β, IL-6, and IL-12 (Figure 6B). Among these differentially expressed genes revealed by transcriptome analysis, the mRNA level of HIF1α was upregulated by p72 (Figure 6C). HIF1α has been reported to regulate the production of IFN-γ and IL-1β [35,36]. Therefore, it is reasonable that p72 could enhance the expression of pro-inflammatory cytokines IFN-γ, IL-1β, IL-6, and IL-12 via the HIF1α pathway. To determine whether p72 regulated HIF1α expression and whether its aa 377–428 participated in the regulation, 293T cells were co-transfected with the plasmids expressing HIF1α and p72 or p72_∆377–428_. As shown in Figure 6D, overexpression of p72 dramatically increased HIF1α expression, while a weaker HIF1α increase pattern was detected for p72_∆377–428_. Under normoxic conditions, HIF1α is continuously degraded, resulting in low basal HIF1α levels [30]. We also investigated whether there was an interaction between p72 and HIF1α to prevent HIF1α degradation, and whether the aa 377–428 of p72 was involved in this interaction. Immunoprecipitation indicated that there was no interaction between p72 and HIF1α (Figure 6E). Further qRT-PCR confirmed that p72 exhibited stronger induction of HIF1α transcription than p72_∆377–428_ in mouse splenocytes and 293T cells (Figure 6F). To analyze how p72 affected HIF1α transcription, we examined the signaling pathways regulated by p72 and found that the PI3K/AKT signaling pathway was enriched (Figure 6A, right panel). The results from RNA-sequencing and qRT-PCR indicate that the transcriptions of PI3K/AKT-related AKT, PDK1, and RPS6 were increased by p72 (Figure 6G,H). PDK1 can activate AKT via phosphorylation at its Thr308, and RPS6 can regulate HIF1α expression [51,52]. Since the PI3K/AKT signaling pathway has been reported to regulate HIF1α transcription [53], we hypothesized that p72 expression would increase HIF1α transcription via the AKT signaling pathway. To test this hypothesis, we examined the impact of p72 and p72_∆377–428_ expression on AKT by immunoblotting. p72 expression increased the level of AKT and phosphorylated AKT proteins, and the deletion of aa 377–428 significantly reduced phosphorylated AKT proteins (Figure 6I). Taken together, these results suggest that the aa 377–428 of p72 participates in the regulation of pro-inflammatory cytokines via the AKT/HIF1α pathway.

## 4. Discussion

The abundant expression and high antigenicity of p72 make it the one focus of ASFV studies. The p72 protein has been engineered to be expressed in multiple systems for its functional study [10,13,54,55]. In this study, we accidentally obtained a mutant p72 protein (p72_∆377–428_) with the short amino acid (377–428) completely lost, which might result from the genetic recombination taking place at nucleotides of “CTTTTTGTA”, namely the nucleotides of 1120–1128 and 1276–1284 of p72 in BL21 (DE3). The structural prediction shows that p72_∆377–428_ is quite different from full-length p72, which has a “compact” structure, as it possesses an “open” structure (Figure 2B,C). The “open” structure might help to induce more antibodies [46]. Future studies should involve a range of experimental approaches (e.g., NMR, cryo-EM) to validate these findings.

Antibodies are believed to play a crucial role in controlling ASFV infection, as the passive transfer of colostrum or serum antibodies from convalescent pigs can lead to reduced viremia and delay the onset of clinical signs and mortality following ASFV challenge [56].

p72-mediated humoral responses have received extensive attention. Accumulating studies have screened p72 antigenic epitopes and their corresponding monoclonal antibodies [13,15,17]. Heimerman et al. mapped the B-cell epitopes of the p72 protein and identified four regions: aa 156–165, aa 265–280, aa 280–294, and aa 294–303 [17]. Later, another eight novel B-cell epitopes were identified for the p72 protein: aa 31–40, aa 41–45, aa 56–63, aa 69–77, aa 185–189, aa 195–205, aa 223–233, aa 249–258, and aa 507–517 [13,15,16]. All these epitopes are linear, and most are consistent across different ASFV strains. The production of antibodies targeting the p72 protein and the identification of its antigenic epitopes can facilitate a deeper understanding of the virus’s invasion mechanism and potentially serve as a foundation for the development of vaccines and diagnostic reagents [57]. The time course of antibody titers elicited by p72 and p72_∆377–428_ reveals similar dynamics of IgG secretions upon immunization with the recombinant proteins. However, the endpoint titer of the antibody induced by p72_∆377–428_ was stronger than that induced by p72 on week 2 and 4 after a second booster immunization (Figure 3B). Multi-epitope and linear-epitope vaccines can serve as promising strategies against African swine fever. We compared the antibody titers of various ASFV vaccines, including multi-epitope vaccines [58] and viral vector vaccines [59,60]. The reported antibody titers for these vaccines generally ranged from 10^4^ to 10^6^, whereas the antibody titer induced by p72_∆377–428_ was within the range of 10^5^ to 10^6^. This suggests that p72_∆377–428_ has the potential to be a vaccine candidate. Moreover, *E. coli*-expressed p72_∆377–428_ can distinguish ASFV-positive swine sera from ASFV-free sera (Figure 3E), implying its potential application as a diagnostic reagent.

Cytokines are secreted proteins that mediate and regulate immunity and inflammation [61]. They have become important diagnostic, prognostic, and therapeutic agents in diseases [62,63]. It is reported that ASFV is related to the elevation of secretion levels of several cytokines in vivo and in vitro [1,64]. Pro-inflammatory cytokines play pivotal roles in all aspects of immune responses, orchestrating the innate and adaptive immune response [19]. Virulent ASFV induced continuous increases in almost all key pro-inflammatory cytokines involved in viral infection [65]. p72 bears T-cell antigenic epitopes which can trigger T cells to secrete pro-inflammatory cytokines once presented by major histocompatibility complexes [49,54,66]. For ASFV infection, IFN-γ is an important pro-inflammatory cytokine [24,26]. Previous reports found that peripheral blood mononuclear cells (PBMCs) from pigs recovered from ASFV infection produced IFN-γ upon stimulation with the virus [67]. IFN-γ secretion was also detected in the co-culture of the lymphocytes from ASFV-immunized pigs and in the p72 peptide, suggesting the contribution of p72 to IFN-γ secretion [49]. Our data show that p72-immunized murine splenocytes secrete more IFN-γ than p72_∆377–428_-immunized murine splenocytes (Figure 4A). This reduction was also phenocopied when comparing p72-expressing 3D4/21 cells with p72_∆377–428_-expressing cells in the context of *Salmonella* infection (Figure 4E). p72_∆377–428_ immunization also triggered reduced secretions of other cytokines including IL-1β, IL-6, and IL-12, in murine splenocytes after stimulation compared to p72 immunization (Figure 5H–J). IL-1β, IFN-γ, IL-6, and IL-12 are pro-inflammatory cytokines that promote the development of protective immune responses. It should be noted that the induction of IL-10 in p72-immunizedmurine splenocytes may be attributed to the induction of IL-12. IL-12 has been reported to induce the expression of its inhibitor IL-10, suggesting that the immune system is equipped with an intrinsic negative feedback mechanism that limits ongoing T-cell activation [68,69,70]. Through comparison with the p72 protein, we found that the p72_∆377–428_ protein reduces the secretion of pro-inflammatory cytokines in mice, showing that the aa 377–428 region was very important for the immune response induced by ASFV p72. In terms of vaccine development, the reduction in cytokine production is a negative outcome, indicating that p72_∆377–428_ potentially has an immunosuppressive effect. It is worth noting that, compared to the control group (PBS group), p72_∆377–428_ still induces a higher level of cytokine production, albeit at a lower level than the wild type (p72 group). p72_∆377–428_ can induce a stronger humoral immune response and lower levels of inflammatory cytokines. This characteristic may contribute to mitigating the cytokine storm associated with ASFV infection. The limitation of this study is that the experiments were conducted in mice rather than in the natural hosts of ASFV, pigs. In the future, a series of critical experiments, including protective efficacy studies and virus neutralization assays, will need to be conducted in pigs. Further investigations are required to verify the p72-related immune response observed in mice. These findings provide novel insights into the immunological function and corresponding mechanism of ASFV p72.

Some cytokines, such as IL-2 and IFNs, are well known for their ability to promote immune and inflammatory responses [19]. However, these cytokines also have crucial immunosuppressive functions [19]. The balance between the pro-inflammatory and immunosuppressive functions of these well-known cytokines contributes to promoting the development of protective immune responses. It should be noted that even if the cellular immune response has been activated, death still may occur [49]. Exaggerated immune activation associated with the excessive release of cytokines, known as a “cytokine storm”, has dangerous consequences [24,28,62]. The following three criteria can be used to identify cytokine storms: elevated circulating cytokine levels, acute systemic inflammatory symptoms, and either secondary organ dysfunction (often renal, hepatic, or pulmonary) due to inflammation beyond that which could be attributed to a normal response to a pathogen (if a pathogen is present), or any cytokine-driven organ dysfunction (if no pathogen is present) [28]. Some results showed that pigs coordinate a defense against the virus in the form of an inflammatory cytokine storm in response to ASFV infection [24,65,71]. An acute ASFV infection-triggered cytokine storm always correlates with host death, which further exacerbates ASF pathogenesis [65]. For the treatment of COVID-19, controlling the inflammatory cytokine storm is an effective means in the absence of specific drugs [49,72,73]. Attention should be paid to the pro-inflammatory cytokines induced by ASFV such as IL-1β, IFN-γ, IL-6, and IL-12. If the cytokine storm produced after ASFV infection is controlled, ASFV-infected pigs could survive [49]. The difference in the pattern of pro-inflammatory cytokines induced by p72 and p72_∆377–428_, implying that p72_∆377–428_ could contribute to reducing the risk of a cytokine storm caused by the excessive release of pro-inflammatory cytokines compared to p72, might assist us in understanding the causes of an ASFV-induced cytokine storm and finding ways to control it.

There is an essential association between hypoxia and inflammation. HIF1α is expressed in several types of innate immune cells, including macrophages, DCs, and Th17 cells [30,74]. The HIF pathway provides these cells with metabolic switches, allowing them to respond appropriately to the significant changes in energy requirements and adapt to the hypoxic conditions that might prevail in inflamed tissue [30]. HRE-containing genes primarily encode proteins that allow for cell adaptation to hypoxic environments. Additionally, there is a whole range of immune-related genes that have HREs in their promoters, including IL-1β and IFN-γ [35,36]. Under normoxic conditions, the deletion of HIF1α reduced the transcriptional levels of IFN-γ in restimulated CD4^+^ T cells, and the HIF1α protein also boosted LPS-induced IL-1β mRNA [35,75]. The transcriptional level of HIF1α increased in mouse splenocytes stimulated by p72 in vitro, but with a smaller increase in the p72_∆377–428_ group. We also obtained a similar result in 293T cells (Figure 6D–F).

The signaling pathways regulating HIF1α under normoxic conditions typically involve the activation of PI3K/AKT signaling and/or MAP kinase signaling [76]. In contrast to the decreased degradation of HIF1α that occurs during hypoxia, PI3K/AKT signal stabilizes and transactivates HIF1α regardless of oxygen levels [77]. Activated PI3K/AKT signaling upregulates HIF1α transcription and translation [77,78]. As an important kinase, AKT is at the crossroads of different signaling pathways and mediates tumor cell proliferation, survival, metabolism angiogenesis, and metastasis [79]. Owing to its importance, the activity of AKT is strictly regulated. It is well known that the phosphorylation of AKT is critical for its activation [52]. In response to an upstream stimulus, PDK1 activates AKT via phosphorylation at its Thr308, and sequential phosphorylation at Ser473 allows for its full activation [51]. Another vital AKT activating pathway is mediated by mTORC2, which interacts with the regulatory hydrophobic domain of AKT to phosphorylate it at Ser473 [80]. Both mTOR and GβL (also known as MLST8) assemble into mTORC2 [81]. It has been reported that GβL was identified as a binding partner of p72 on the surface of ASFV virions by using an IP-MS assay [82]. Here, we have demonstrated that p72 expression in 293T cells boosts HIF1α expression, at least in part, by increasing AKT expression and phosphorylation to activate the AKT pathway, but with a smaller increase in the p72_∆377–428_ group (Figure 6G–I). This suggests that p72_∆377–428_ may regulate the expression of HIF1α through the AKT pathway. Future studies will need to employ experimental approaches, such as phosphor-proteomics, pathway inhibition, and gene silencing, to validate these findings.

In conclusion, we discovered that p72_∆377–428_ recognized an antiserum against ASFV. p72_∆377–428_ elicited robust humoral immunity in mice and reduced the level of pro-inflammatory cytokines including IFN-γ, IL-1β, IL-6, and IL-12, thus possibly attenuating the risk of a cytokine storm, compared to p72. The AKT/HIF1α signaling pathway is involved in the reduced induction of pro-inflammatory cytokines by p72_∆377–428._ These data indicate that p72_∆377–428_ could serve as a novel candidate for ASFV vaccines and diagnostic reagents, and can contribute to eliminating our insufficient understanding of the interaction of ASFV with the host immune system.

## Data Availability

The datasets used and/or analyzed in this study are available from the corresponding authors upon reasonable request.

## Supplementary Materials

The following supporting information can be downloaded at: https://www.mdpi.com/article/10.3390/v17020194/s1. Table S1: Settings and functional links of the online software, Table S2: The primer sets were used for Plasmid construction, Table S3: The primer sets were used for real-time qPCR analysis, Table S4: Epitope Prediction Results table of p72, Table S5: Epitope Prediction Results table of p72_∆377-428_, Table S6: T-cell epitopes with H-2Dd as the representative MHC molecule of the p72 protein, Table S7: T-cell epitopes with SLA-0101 as the representative MHC molecule of the p72 protein.
