# Supplementary material for: A Mutant of Africa Swine Fever Virus Protein p72 Enhances Antibody Production and Regulates the Production of Cytokines
Source: Viruses. 2025 Jan 30;17(2):194. doi: 10.3390/v17020194 (PMC11860850; doi:10.3390/v17020194)
Supplement: Supplementary file 1 [file viruses-17-00194-s001.zip › viruses-3393960-supplementary.pdf]

Supplementary Materials

Supplementary Table S1. Settings and functional links of the online software

Software	Functional links	Method	Setting
IEDB	B Cell Epitope Prediction: http://tools.iedb.org/bcell/	Bepipred Linear Epitope Prediction 2.0	Threshold > 0.55
	T Cell Epitope Prediction: http://tools.iedb.org/mhci/	NetMHCpan 4.1	Specie: mouse/pig; Allele: H-2Dd/SLA-0101; Length: All
SWISS-MODEL	https://swissmodel.expasy.org/	SWISS-MODEL	GMQE > 0.7

Supplementary Table S2. The primer sets were used for Plasmid construction.

Gene	Sequence
pET-72-F	CGCGGATCCATGGCATCA
pET-72-R	CCGCTCGAGGGTACTGTAA
pCAGGS-HA-72-F	CCGGAATTCATGGCATCAGGAGGAGCTT
pCAGGS-HA-72-R	GGAAGATCTGTGGTGATGGTGATGATGGGTACTGTAAC
B602L-F	ATGGCAGAATTTAATATTGA
B602L-R	TTACAATTCTGCTTTTGTATA
pCAGGS-HA-HIF1A-F	CGCGGATCCATGGAAGGCGCCG
pCAGGS-HA-HIF1A-F	CCGCTCGAGTCAGTTAACTTGATCCAAAGCTCTGAG
pCDNA3.1-Flag-HIF1α-F	CCGCTCGAGATGGAAGGCGCCGGC
pCDNA3.1-Flag-HIF1α-F	CGGGGTACCTTGTTAACTTGATCCAAAGCTCTGAG

The restriction endonuclease sites are emphasized in yellow.

Supplementary Table S3. The primer sets were used for real-time qPCR analysis.

Gene	Sequence
(Mouse)IL-1β-F	TCAGGCAGGCAGTATCACTC
(Mouse)IL-1β-R	CTAATGGGAACGTCACACACC
(Mouse)IL-4-F	GGTCTCAACCCCCAGCTAGT
(Mouse)IL-4-R	GCCGATGATCTCTCTCAAGTGAT
(Mouse)IL-6-F	TGGTCTTCTGGAGTACCATAGC
(Mouse) IL-6-R	TGTGACTCCAGCTTATCTCTTGG
(Mouse)IL-10-F	GGAGCATTTGAATTCCCTGGGT
(Mouse)IL-10-R	TAGACACCTTGGTCTTGGAGCTTAT
(Mouse) IL-12 p35- F	CCTCAGTTTGGCCAGGGTC
(Mouse) IL-12 p35-R	CAGGTTTCGGGACTGGCTAAG

(Mouse) IL-12 p40- F	GGAAGCACGGCAGCAGAATA
(Mouse) IL-12 p40-R	AACTTGAGGGAGAAGTAGGAATGG
(Mouse)IFN-γ-F	AGGAACTGGCAAAAGGATGGT
(Mouse)IFN-γ-R	AATGACGCTTATGTTGTTGCTGA
(Mouse)HIF1α-F	TGGACTTGTCTCTTTCTCCGC
(Mouse)HIF1α-R	CGACGTTCAGAACTCATCCTATTTT
(Human)HIF1α-F	TTTTGGCAGCAACGACACAG
(Human)HIF1α-R	GCGTTTCAGCGGTGGGTAAT
(Human)PDK1-F	CGTTCAATTGGTACAAAGCTGGTAT
(Human)PDK1-R	TGCAGGCCATACAGCATAAAC
(Human)RPS6-F	AAGCTGAACATCTCCTTCCCA
(Human)RPS6-R	ATTCGGACCACATAACCCTTC
(Human)AKT-F	CCACACACTCACCGAGAACC
(Human)AKT-R	ACAGGTGGAAGAACAGCTCG
(Human)profilin1-F	ACGCCTACATCGACAACCTC
(Human)profilin1-R	CCTCAGCTGGCGTGATGT
(Mouse)β-actin- F	GCCGCCAGCTCACCATGGATGAT
(Mouse)β-actin- R	ATGCCCACCATCACGCCCTGG

Supplementary Table S4. Epitope Prediction Results table of p72

No.	Start	End	Peptide	Length
1	31	52	SNIKNVNKSYGKPDPEPTLSQI	22
2	65	91	KPYVPVGFEYNKVRPHTGTPTLGNKLT	27
3	116	151	SWQDAPIQGTSQMGAHGQLQTFPRNGYDWDNQTPLE	36
4	160	172	PFGRPIVPGTKNA	13
5	185	189	ERLYE	5
6	193	206	FDVNGNSLDEYSSD	14
7	216	237	IPGDKMTGYKHLVGQEVSVEGT	22
8	247	308	DLHKPHQSKPILTDENDTQRTCSHTNPKFLSQHFPENSHNI QTAGKQDITPITDATYLDIRR	62
9	311	324	HYSCNGPQTPKYYQ	14
10	340	344	NVNLA	5
11	364	370	QKDLVNEF	8
12	380	393	RFIAGRPSRRNIRF	14
13	443	457	VTHTNNNHHDEKLMS	15
14	473	489	TWNISDQNPHQHRDWHK	17
15	498	528	MQPTHHAEISFQDRDTALPDACSSISDISPV	31
16	552	553	KF	2
17	555	557	SKF	3
18	563	574	PFHYGGNAIKTPD	13
19	589	601	REEYQPSGHINVS	13
20	613	616	DYVG	4

The epitopes located in the AA 377-428 are emphasized in yellow.

Supplementary Table S5. Epitope Prediction Results table of p72$_{\Delta 377\text{-}428}$

No.	Start	End	Peptide	Length
1	31	52	SNIKNVNKSYGKPDPEPTLSQI	22
2	65	91	KPYVPVGFEYNKVRPHTGTPTLGNKLT	27
3	116	151	SWQDAPIQGTSQMGAHGQLQTFPRNGYDWDNQTPLE	36
4	160	171	PFGRPIVPGTKN	12
5	185	189	ERLYE	5
6	193	206	FDVNGNSLDEYSSD	14
7	217	236	PGDKMTGYKHLVGQEVSVEG	20
8	248	304	LHKPHQSKPILTDENDTQRTCSHTNPKFLSQHFPENSHNIQ TAGKQDITPITDATYL	57
9	313	324	SCNGPQTPKYYQ	12
10	341	343	VNL	3
11	364	371	QKDLVNEF	8
12	391	405	VTHTNNNHHDEKLMS	15
13	421	436	TWNISDQNPHQHRDWH	16
14	446	475	MQPTHHAEISFQDRDTALPDACSSISDISP	30
15	500	501	KF	2
16	503	505	SKF	3
17	511	523	PFHYGGNAIKTPD	13
18	537	549	REEYQPSGHINVS	13
19	561	564	DYVG	4

Supplementary Table S6 T-cell epitopes with H-2Dd as the representative MHC molecule of the p72 protein.

allele	start	end	length	peptide	score	Rank (%)
H-2-Dd	419	427	9	VTPEIHNLF	0.456899	0.01
H-2-Dd	419	426	8	VTPEIHNL	0.297504	0.02
H-2-Dd	525	534	10	ISPVTYPITL	0.248964	0.03
H-2-Dd	498	506	9	MQPTHHAEI	0.143214	0.09
H-2-Dd	348	356	9	VSIPFGERF	0.142439	0.09
H-2-Dd	323	330	8	YQPPLALW	0.141939	0.09
H-2-Dd	40	49	10	YGKPDPEPTL	0.141321	0.09
H-2-Dd	82	90	9	GTPTLGNKL	0.122719	0.1
H-2-Dd	238	245	8	SGPLLCNI	0.12141	0.1
H-2-Dd	627	635	9	ASAINFLLL	0.118677	0.11
H-2-Dd	525	532	8	ISPVTYPI	0.115313	0.11
H-2-Dd	81	90	10	TGTPTLGNKL	0.10695	0.13
H-2-Dd	602	610	9	RAREFYISW	0.097359	0.14
H-2-Dd	271	280	10	TNPKFLSQHF	0.087174	0.18
H-2-Dd	323	331	9	YQPPLALWI	0.080486	0.2
H-2-Dd	592	600	9	YQPSGHINV	0.077701	0.21
H-2-Dd	625	633	9	VSASAINFL	0.07648	0.22
H-2-Dd	279	287	9	HFPENSHNI	0.069839	0.25
H-2-Dd	238	248	11	SGPLLCNIHDL	0.06929	0.25
H-2-Dd	92	101	10	FGIPQYGDFF	0.066299	0.26
H-2-Dd	38	49	12	KSYGKPDPEPTL	0.06344	0.29
H-2-Dd	349	356	8	SIPFGERF	0.057971	0.3
H-2-Dd	572	580	9	KTPDDPGAM	0.056488	0.31
H-2-Dd	418	427	10	FVTPEIHNLF	0.056037	0.31
H-2-Dd	468	476	9	IGLKPTWNI	0.055593	0.31
H-2-Dd	545	553	9	HGINLIDKF	0.054516	0.32
H-2-Dd	349	359	11	SIPFGERFITI	0.052899	0.33
H-2-Dd	572	582	11	KTPDDPGAMMI	0.050699	0.37
H-2-Dd	344	352	9	AIPSVSIPF	0.048571	0.39
H-2-Dd	147	157	11	QTPLEGAVYTL	0.047208	0.41
H-2-Dd	82	92	11	GTPTLGNKLTF	0.046554	0.41
H-2-Dd	295	304	10	ITPITDATYL	0.045359	0.43
H-2-Dd	389	397	9	RNIRFKPWF	0.044461	0.44
H-2-Dd	315	323	9	NGPQTPKYY	0.04362	0.46
H-2-Dd	295	303	9	ITPITDATY	0.041907	0.48
H-2-Dd	419	428	10	VTPEIHNLFV	0.041436	0.48
H-2-Dd	628	635	8	SAINFLLL	0.04101	0.49
H-2-Dd	529	537	9	TYPITLPII	0.039671	0.51
H-2-Dd	420	427	8	TPEIHNLF	0.038204	0.53

H-2-Dd	397	405	9	FIPGVINEI	0.037545	0.54
H-2-Dd	479	487	9	QNPHQHRDW	0.037252	0.54
H-2-Dd	572	581	10	KTPDDPGAMM	0.036532	0.56
H-2-Dd	321	329	9	KYYQPPLAL	0.036284	0.56
H-2-Dd	417	427	11	LFVTPEIHNLF	0.036017	0.57
H-2-Dd	203	210	8	YSSDVTTL	0.034905	0.58
H-2-Dd	627	634	8	ASAINFLL	0.03353	0.61
H-2-Dd	528	536	9	VTYPITLPI	0.032843	0.62
H-2-Dd	598	606	9	INVSRAREF	0.030548	0.68
H-2-Dd	318	327	10	QTPKYYQPPL	0.030445	0.69
H-2-Dd	80	90	11	HTGTPTLGNKL	0.030117	0.7
H-2-Dd	389	396	8	RNIRFKPW	0.029687	0.7
H-2-Dd	323	333	11	YQPPLALWIKL	0.029648	0.7
H-2-Dd	526	534	9	SPVTYPITL	0.029254	0.71
H-2-Dd	78	86	9	RPHTGTPTL	0.02919	0.71
H-2-Dd	64	72	9	FKPYVPVGF	0.028962	0.72
H-2-Dd	349	357	9	SIPFGERFI	0.02881	0.72
H-2-Dd	549	557	9	LIDKFPSKF	0.028313	0.74
H-2-Dd	460	468	9	KWPIEYMFI	0.027646	0.76
H-2-Dd	352	359	8	FGERFITI	0.027402	0.77
H-2-Dd	418	426	9	FVTPEIHNL	0.027269	0.78
H-2-Dd	93	101	9	GIPQYGDFF	0.02673	0.8
H-2-Dd	625	632	8	VSASAINF	0.025975	0.82
H-2-Dd	385	393	9	RPSRRNIRF	0.025969	0.82
H-2-Dd	39	49	11	SYGKPDPEPTL	0.025946	0.82
H-2-Dd	497	506	10	IMQPTHHAEI	0.025742	0.83
H-2-Dd	524	534	11	DISPVTYPITL	0.025178	0.85
H-2-Dd	70	77	8	VGFEYNKV	0.02498	0.85
H-2-Dd	322	330	9	YYQPPLALW	0.024787	0.86
H-2-Dd	41	49	9	GKPDPEPTL	0.024474	0.87
H-2-Dd	318	329	12	QTPKYYQPPLAL	0.023764	0.91
H-2-Dd	498	508	11	MQPTHHAEISF	0.023692	0.91
H-2-Dd	42	49	8	KPDPEPTL	0.023407	0.93
H-2-Dd	363	371	9	SQKDLVNEF	0.022717	0.96
H-2-Dd	277	287	11	SQHFPENSHNI	0.022627	0.96

The epitopes located in the AA 377-428 are emphasized in yellow.

Supplementary Table S7 T-cell epitopes with SLA-0101 as the representative MHC molecule of the p72 protein.

allele	start	end	length	peptide	score	Rank (%)
SLA-1*0101	50	58	9	SQIEETHLV	0.563398	0.03
SLA-1*0101	22	30	9	AQDLLNSRI	0.503763	0.05
SLA-1*0101	522	530	9	ISDISPVTY	0.430445	0.1
SLA-1*0101	573	581	9	TPDDPGAMM	0.407028	0.11
SLA-1*0101	203	211	9	YSSDVTTLV	0.38763	0.14
SLA-1*0101	42	49	8	KPDPEPTL	0.378981	0.15
SLA-1*0101	572	581	10	KTPDDPGAMM	0.374252	0.16
SLA-1*0101	78	86	9	RPHTGTPTL	0.363008	0.17
SLA-1*0101	157	165	9	LVDPFGRPI	0.366648	0.17
SLA-1*0101	199	207	9	SLDEYSSDV	0.371778	0.17
SLA-1*0101	418	426	9	FVTPEIHNL	0.36266	0.17
SLA-1*0101	549	557	9	LIDKFPSKF	0.351197	0.19
SLA-1*0101	142	150	9	YDWDNQTPL	0.33878	0.21
SLA-1*0101	192	200	9	RFDVNGNSL	0.306583	0.28
SLA-1*0101	626	634	9	SASAINFLL	0.299591	0.3
SLA-1*0101	526	534	9	SPVTYPITL	0.290035	0.33
SLA-1*0101	42	52	11	KPDPEPTLSQI	0.282066	0.35
SLA-1*0101	508	515	8	FQDRDTAL	0.274473	0.37
SLA-1*0101	120	128	9	APIQGTSQM	0.273974	0.38
SLA-1*0101	363	371	9	SQKDLVNEF	0.267239	0.4
SLA-1*0101	592	600	9	YQPSGHINV	0.265308	0.41
SLA-1*0101	101	109	9	FHDMVGHHI	0.257435	0.44
SLA-1*0101	179	187	9	YCEYPGERL	0.258208	0.44
SLA-1*0101	625	633	9	VSASAINFL	0.258941	0.44
SLA-1*0101	498	506	9	MQPTHHAEI	0.25118	0.48
SLA-1*0101	573	582	10	TPDDPGAMMI	0.250177	0.48
SLA-1*0101	419	427	9	VTPEIHNLF	0.24761	0.49
SLA-1*0101	97	105	9	YGDFFHDMV	0.243302	0.51
SLA-1*0101	204	211	8	SSDVTTLV	0.243472	0.51
SLA-1*0101	521	530	10	SISDISPVTY	0.244698	0.51
SLA-1*0101	496	504	9	AIMQPTHHA	0.242578	0.52
SLA-1*0101	298	306	9	ITDATYLDI	0.238513	0.53
SLA-1*0101	199	210	12	SLDEYSSDVTTL	0.233698	0.55
SLA-1*0101	126	134	9	SQMGAHGQL	0.223323	0.6
SLA-1*0101	348	356	9	VSIPFGERF	0.223066	0.6
SLA-1*0101	619	627	9	TTADLVVSA	0.223327	0.6
SLA-1*0101	203	210	8	YSSDVTTL	0.214666	0.64
SLA-1*0101	1	9	9	MASGGAFCL	0.21302	0.65
SLA-1*0101	50	57	8	SQIEETHL	0.207812	0.69
SLA-1*0101	476	484	9	ISDQNPHQH	0.204947	0.7

SLA-1*0101	541	549	9	SVTAHGINL	0.200631	0.75
SLA-1*0101	572	582	11	KTPDDPGAMMI	0.196918	0.78
SLA-1*0101	634	642	9	LLQNGSAVL	0.197278	0.78
SLA-1*0101	397	405	9	FIPGVINEI	0.195616	0.79
SLA-1*0101	514	522	9	ALPDACSSI	0.19578	0.79
SLA-1*0101	323	331	9	YQPPLALWI	0.194174	0.8
SLA-1*0101	528	536	9	VTYPITLPI	0.194291	0.8
SLA-1*0101	233	241	9	SVEGTSGPL	0.19332	0.81
SLA-1*0101	520	528	9	SSISDISPV	0.192684	0.81
SLA-1*0101	102	110	9	HDMVGHHIL	0.19069	0.82
SLA-1*0101	233	242	10	SVEGTSGPLL	0.191036	0.82
SLA-1*0101	11	19	9	ANDGKADKI	0.189151	0.84
SLA-1*0101	404	412	9	EISLTNNEL	0.188031	0.85
SLA-1*0101	627	635	9	ASAINFLLL	0.188208	0.85
SLA-1*0101	342	350	9	NLAIPSVSI	0.184512	0.89
SLA-1*0101	296	304	9	TPITDATYL	0.180045	0.94
SLA-1*0101	234	242	9	VEGTSGPLL	0.171943	1

The epitopes located in the AA 377-428 are emphasized in yellow.
